# Inhibition of Brain Area and Functional Connectivity in Idiopathic Sudden Sensorineural Hearing Loss With Tinnitus, Based on Resting-State EEG

**DOI:** 10.3389/fnins.2019.00851

**Published:** 2019-08-14

**Authors:** Yuexin Cai, Jiahong Li, Yanhong Chen, Wan Chen, Caiping Dang, Fei Zhao, Wenrui Li, Guisheng Chen, Suijun Chen, Maojin Liang, Yiqing Zheng

**Affiliations:** ^1^Department of Otolaryngology, Sun Yat-sen Memorial Hospital, Sun Yat-sen University, Guangzhou, China; ^2^Institute of Hearing and Speech-Language Science, Sun Yat-sen University, Guangzhou, China; ^3^State Key Laboratory of Ophthalmology, Zhongshan Ophthalmic Center, Sun Yat-sen University, Guangzhou, China; ^4^Affiliated Brain Hospital of Guangzhou Medical University, Guangzhou, China; ^5^Department of Psychology, Guangzhou Medical University, Guangzhou, China; ^6^Department of Speech Language Therapy and Hearing Science, Cardiff Metropolitan University, Cardiff, United Kingdom; ^7^Department of Hearing and Speech Science, Xinhua College, Sun Yat-sen University, Guangzhou, China

**Keywords:** tinnitus, ISSNHL, EEG, source localization, functional connectivity

## Abstract

This study aimed to identify the mechanism behind idiopathic sudden sensorineural hearing loss (ISSNHL) in patients with tinnitus by investigating aberrant activity in areas of the brain and functional connectivity. High-density electroencephalography (EEG) was used to investigate central nervous changes in 25 ISSNHL subjects and 27 healthy controls. ISSNHL subjects had significantly reduced activity in the left frontal lobe at the alpha 2 frequency band compared with controls. Linear lagged connectivity and lagged coherence analysis showed significantly reduced functional connectivity between the temporal gyrus and supramarginal gyrus at the gamma 2 frequency band in the ISSNHL group. Additionally, a significantly reduced functional connectivity was found between the central cingulate gyrus and frontal lobe under lagged phase synchronization analysis. These results strongly indicate inhibition of brain area activity and change in functional connectivity in ISSNHL with tinnitus patients.

## Introduction

Sudden sensorineural hearing loss (SSNHL) defines rapid onset hearing loss with reduction in hearing of no less than 30 dB in at least three consecutive audiometric frequencies within 3 days (Stachler et al., 2012). SSNHL is typically accompanied by sudden onset of tinnitus, vertigo and ear fullness (Koç and Sanisoğlu, 2003). The incidence of SSNHL ranges from 3.9 to 27.5 people per 100,000/year (Nosrati-Zarenoe et al., 2010). The etiology of more than two thirds of SSNHL is unclear and considered idiopathic (Chau et al., 2010). Clinicians employ multiple treatment options to cure idiopathic SSNHL (ISSNHL), including oral and transtympanic steroid therapy (Kuhn et al., 2011). However, the therapeutic effectiveness between ISSNHL patients is highly variable. Unfortunately, some ISSNHL subjects do not improve even with high quality treatment, and indeed there may be some adverse effects (Kuhn et al., 2011). The therapeutic effectiveness of ISSNHL treatments may be improved were a better understanding of the mechanism behind ISSNHL available.

Tinnitus, a common and troublesome symptom of ISSNHL, is the perception of sound that lacks any external or internal sound source (Kang et al., 2017). The incidence of tinnitus in ISSNHL patients is between 80 and 95% (Rah et al., 2015; Nogueira-Neto et al., 2016). Tinnitus brings a series of adverse effects to ISSNHL patients, including insomnia and depression (Chen et al., 2013). Additionally, tinnitus symptoms often continue in ISSNHL patients with tinnitus even after treatment, indicating that these patients may have aberrant changes in the brain. Therefore, it is advisable that clinicians pay more attention to the treatment of tinnitus when acting to cure ISSNHL patients.

To treat ISSNHL and tinnitus subjects effectively requires an in-depth investigation into the underlying mechanism of ISSNHL. Current research shows abnormal brain area activity and network connectivity in ISSNHL patients. For example, Micarelli et al. (2017) showed that ISSNHL subjects had hypermetabolism in the right superior and medial frontal gyrus, and a relative reduction in fluorodeoxyglucose uptake in the right middle temporal, precentral and postcentral gyrus, left posterior cingulate cortex, left lingual, superior, middle temporal and middle frontal gyrus and left insula. A study by Xu et al. (2016) showed that SSNHL subjects had some increased activities between auditory and non-auditory networks, including the default mode network (DMN) and limbic system. Other studies have explored the intrinsic mechanism of chronic tinnitus with hearing impairment. It is generally believed that the generation and development of tinnitus is not only related to peripheral hearing impairment, but also correlated with abnormal neural activity (Chen et al., 2014, 2015a, 2016, 2017). A functional imaging study by Lanting et al. (2009) suggested that tinnitus patients had hyperactivity in the auditory system. Tinnitus subjects also show some aberrant neural activity in non-auditory regions, including the frontal gyrus, limbic system, cerebellum and insula (Chen et al., 2017). A number of studies have identified abnormal brain networks in tinnitus including the auditory network (Leaver et al., 2016), DMN (Leaver et al., 2016), dorsal attention network (Schmidt et al., 2013), ventral attention network (Burton et al., 2012) and visual system (Chen et al., 2015b). However, there are few studies exploring the difference of brain area and network connectivity between ISSNHL patients and healthy subjects. Therefore, our research aimed to investigate the underlying mechanism of ISSNHL and tinnitus based on resting-state electroencephalography (EEG).

Approximately 85% of tinnitus patients have an uninterrupted perception of tinnitus sound (Schecklmann et al., 2014). Resting state brain function measurement is therefore suitable for exploring the neural mechanism of tinnitus and ISSNHL. EEG investigates instantaneous electric activity of the brain by recording electrical potentials with electrodes placed on the scalp. It is a technique that is non-invasive and has high temporal resolution (Ingber and Nunez, 2011). At present, a number of methods can be used to extract useful information from multichannel EEG signals, including spectrum analysis, source localization and neural network function analysis. These analytical methods are useful to explore topological property alterations of the brain.

It is still unknown as to whether neural activity changes occur in ISSNHL patients with tinnitus. Thus, our study aim was to gain brain region activity and functional connectivity of central networks in ISSNHL and tinnitus patients based on resting-state EEG. Moreover, we explored the change in balance between excitation and inhibition of the whole brain in these patients.

## Materials and Methods

### Participants

ISSNHL patients with tinnitus were recruited from the Ear, Nose and Throat clinic, Sun Yat-sen Memorial hospital, Guangzhou, China. Selection criteria for ISSNHL subjects with tinnitus were as follows:

(a) Patients had no less than 30 dB sensorineural hearing impairment associated with tinnitus in more than three consecutive audiometric frequencies that had onset within 3 days.
(b) Patients had sought clinical help due to hearing impairment and tinnitus.
(c) Subjects with middle ear surgery, pulsatile tinnitus, Ménière’s disease, autoimmune hearing loss, acoustic neurinoma, central nervous system disorders, head trauma, depression or insomnia were excluded.

Data was collected before patients received any therapeutic intervention within 30 days. In this study, 25 ISSNHL subjects with tinnitus (9 males and 16 females; age mean = 46.16 years, *SD* = 13.15 years) and 27 healthy subjects (10 males and 17 females; age mean = 41.48 years, *SD* = 13.53 years) were included. All participants were told about the background and purpose of the study. A written consent form was signed by all subjects. This research was approved by the Institution Review Board of the Sun Yat-sen Memorial Hospital at Sun Yat-sen University of China.

### Audiological Examinations

All participants underwent otoscopy and pure tone audiometry. Subjects were asked to sit in an acoustically shielded room for the pure tone audiometry test. Hearing thresholds of the subjects were then measured. Air conduction thresholds at 0.125, 0.25, 0.5, 1, 2, 4, and 8 kHz were measured on both ears. Bone conduction thresholds at 0.25, 0.5, 1, 2, and 4 kHz were also determined. Mean hearing threshold was calculated from hearing thresholds at 0.5, 1, 2, and 4 kHz (British Society of Audiology Recommendation, 1988; Dispenza et al., 2011; Bing et al., 2018).

### Tinnitus Specific Assessments

Information about the duration and laterality of tinnitus was collected. A tinnitus pitch matching test was then performed (Teismann et al., 2011). An approximate pitch match was determined using the frequencies 0.125, 0.25, 0.5, 1, 2, 3, 4, 6, and 8 kHz. Patients were asked to compare the matching pitch and perceived tinnitus pitch; the matching pitch was adjusted based on the feedback from the subject. A more accurate matching pitch was obtained by adjusting the frequency in half-octave steps. Narrow band noise was used if a matching pure tone could not be found. After finding the best matching pitch, the initial level was set to 5 dB above the audiometric threshold to find an approximation of the tinnitus loudness. Loudness was adjusted in 1 dB steps until the patient expressed a loudness match. Tinnitus loudness was computed by subtracting the presented sound intensity level with the auditory threshold at that frequency. Finally, tinnitus severity was evaluated using a Tinnitus Handicap Inventory (THI). The THI used an increasing handicap scale from 0 to 100 to assess the level of handicap (Newman et al., 1996). Patients were asked to fill in the THI before the experiment commenced.

### Data Collection

Electroencephalography signals were gained using a standard procedure. EEG signals were recorded using an EGI 128 channel system. Before the experiment, participants were informed of the aim of this study to ensure they were in a relaxed state. Participants sat in an acoustic and electrically shielded room. Their eyes were open and they were asked to view a computer screen, to blink as little as possible, not to move their head and to keep awake. A mesh electrode cap was positioned with a careful positioning of the center electrode Vref. Impedances of all electrodes were kept at no more than 50 kΩ. The recording system synchronized the EEG data, and the recording lasted for 7 min.

### Preprocessing of EEG Data

Recordings were analyzed in MATLAB for R2013a using EEGLAB for v13.0.0. The whole process was a seven-step instruction as follows. (1) The sampling rate was reduced to 500 Hz. (2) ERPLAB filter was used for sag filtering to remove 50 Hz power frequency interference and the data was band-pass filtered (0.01 Hz high-pass and 100 Hz low-pass). (3) The average reference of all electrodes was used. (4) EEG data was removed with a wide range of drift periods. If the electrode signal was unstable, linear interpolation was performed on the electrode. (5) Independent component analysis algorithm (ICA) was applied to remove independent components related to artifacts. (6) The EEG data was segmented (divided into 2 s). If the amplitude of a segment at any one electrode exceeded 75 μV, then the segment would be removed. (7) Average Fourier cross-spectral matrices were computed for frequency bands, including delta (0.5–3.5 Hz), theta (4–7.5 Hz), alpha 1 (8–10 Hz), alpha 2 (10–12 Hz), beta 1 (13–18 Hz), beta 2 (18.5–21 Hz), beta 3 (21.5–30 Hz), gamma 1 (30.5–44 Hz), and gamma 2 (55–100 Hz).

### Data Analysis

#### Source Localization

Standardized low-resolution brain electromagnetic tomography (sLORETA) was applied to analyze the activation of brain regions in each group and understand the activation distribution of central brain regions in ISSNHL patients with tinnitus in the nine frequency bands mentioned above. After performing a common mean reference transformation (Pascual-Marqui, 2002), the sLORETA algorithm was applied. Electrical neuronal activity was calculated by sLORETA as current density (A.m^–2^). The LORETA-K**e**y software was used to analyze the solution space applied in the research and correlated lead field matrix. The software revisited realistic electrode coordinates (Jurcak et al., 2007) and the lead field (Fuchs et al., 2002) applying the boundary element on the MMI-152 (Montreal Neurological Institute, Canada). According to probabilities returned by the Demon Atlas, the neocortical MNI-152 volume in 6239 voxels was divided and labeled by the sLORETA-Key anatomical template (Lancaster et al., 2000). The co-registration used the correct translation from the MNI-152 space into the Talairach and Tournoux space.

#### Functional Connectivity Analysis

Cross-talking between areas contributing to the source activity can be used to interpret the lagged phase coherence between two brain areas (Congedo et al., 2010). When two brain areas oscillate coherently with a phase lag, cross-talk can be interpreted as information sharing by axonal transmission. Precisely, the data were decomposed in a limited number of cosine and sine waves at the Fourier frequencies by using the discrete Fourier transform. The work of Pascual-Marqui (2007a, b) showed the significant threshold for a given lagged phase coherence value based on asymptotic results. We used sLORETA to extract current density for regions of interest with time series. Power in all 6,239 voxels was normalized to a power of 1 and log transformed at each time point. Therefore, region of interest values reflect the log transformed fraction of total power across all voxels for specific frequencies. As shown in Table 1, regions of interest were determined according to previous studies and the result of source localization (Raichle, 2011).

**TABLE 1:** The regions of interest.

**Brain region**	***X***	***Y***	***Z***
Posterior cingulate	0	–52	27
Medial prefrontal	–1	54	27
Left lateral parietal	–46	–66	30
Right lateral parietal	49	–63	33
Left inferior temporal	–61	–24	–9
Right inferior temporal	0	–12	9
Medial dorsal thalamus	–25	–81	–33
Right posterior cerebellum	25	–81	–33
Left frontal eye field	–29	–9	54
Right frontal eye field	29	–9	54
Left posterior intraparietal sulcus	–26	–66	48
Right posterior intraparietal sulcus	26	–66	48
Left anterior intraparietal sulcus	–44	–39	45
Right anterior intraparietal sulcus	41	–39	45
Left middle temporal	–50	–66	–6
Right middle temporal	53	–63	–6
Left Visual system	–7	83	2
Right Visual system	7	83	2
Left Auditory system	–62	–30	12
Right Auditory system	59	–27	15

### Statistical Analysis

To identify potential differences in resting-state ongoing cortical oscillatory activity between ISSNHL subjects and normal controls, non-parametric statistical analysis of LORETA-KEY images (statistical non-parametric mapping; SnPM) were applied to every contrast using LORETA-KEY’s built-in voxel-wise randomization tests (5000 permutations). We employed at statistic for independent groups with a threshold of *P* < 0.01 (corrected for multiple comparison). A relatively strict threshold for statistical significance was applied as the number of subjects was relatively small. A correction for multiple comparisons in SnPM using 5000 random permutations has been shown to yield similar results with those acquired from a statistical parametric mapping approach using a general linear model with multiple comparisons corrections.

We compared lagged connectivity differences between two groups and used the t-statistics for independent groups with a threshold of *P* < 0.05. Additionally, LORETA-KEY’s built-in voxel-wise randomization tests were used to correct for multiple comparisons for all the voxels included in the region of interest for connectivity analysis (5000 permutations).

## Results

### Source Localization

**FIGURE 1:**
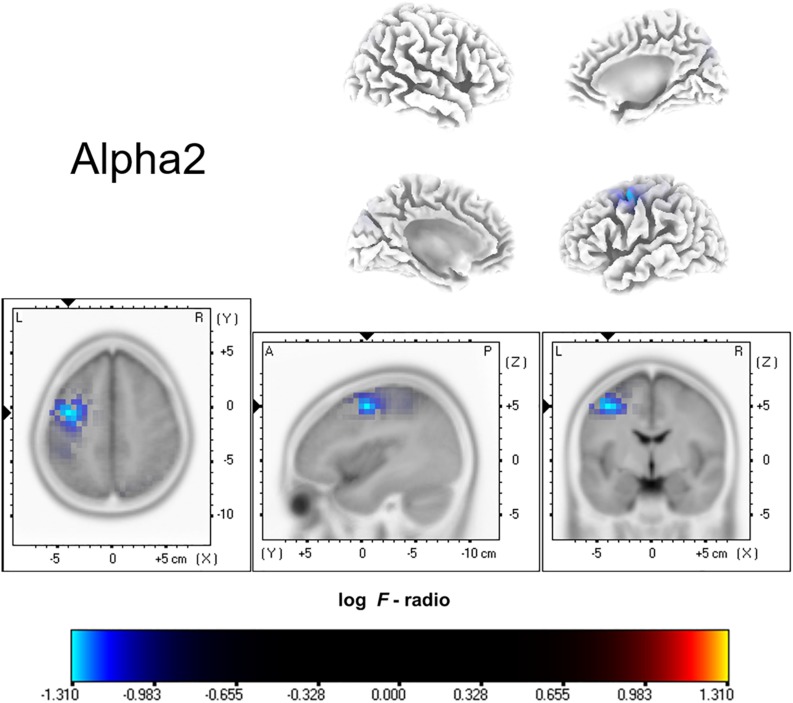
Standardized low-resolution brain electromagnetic tomography. Compared with the healthy group, the ISSNHL group had significantly decreased activity in the left frontal lobe for the alpha 2 frequency band. Labels L, R, A and P represent left, right, anterior and posterior, respectively. Labels S and I represent superior and inferior.

As shown in Figure 1, compared with healthy controls, ISSNHL subjects demonstrated significantly reduced electric activity in the left frontal lobe (BA6; *P* < 0.05) alpha 2 frequency band. In other frequency bands, no significant difference was found between the two groups. Moreover, there was no significant difference in other brain areas.

### Functional Connectivity

**FIGURE 2:**
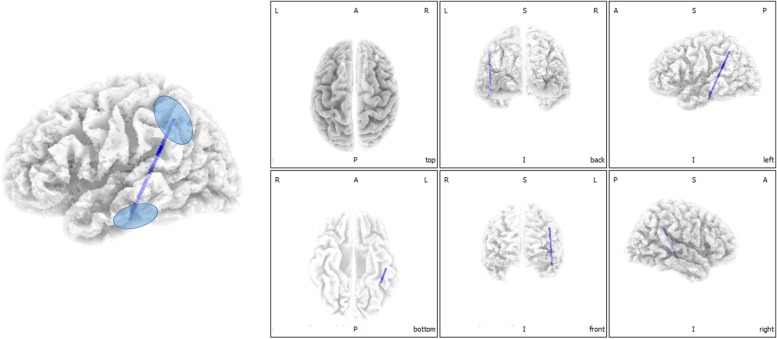
Decreased functional connectivity between temporal gyrus and supramarginal gyrus for the gamma 2 frequency band in the linear lagged connectivity and lagged coherence analysis for ISSNHL patients with tinnitus. Labels L, R, A and P represent the left, right, anterior and posterior, respectively.

**FIGURE 3:**
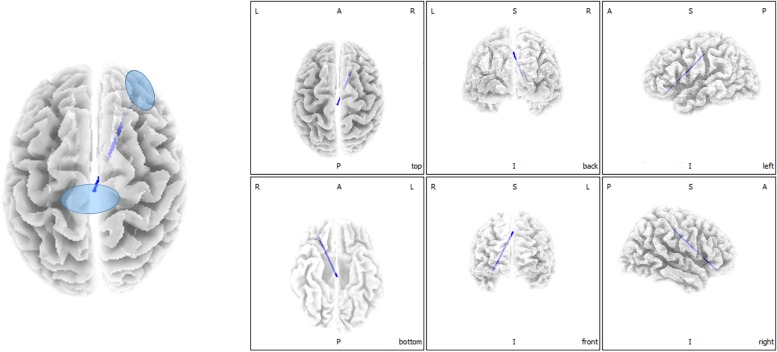
Decreased functional connectivity between central cingulate gyrus and right frontal lobe for gamma 2 frequency band in the lagged phase analysis for ISSNHL patients with tinnitus. Labels L, R, A and P represent the left, right, anterior and posterior, respectively.

Functional connectivity analysis suggested significant differences between ISSNHL subjects and controls in the gamma 2 frequency band. There was significantly decreased functional connectivity between the temporal gyrus (BA20) and supramarginal gyrus (BA40) in the gamma 2 frequency band in the linear lagged connectivity and lagged coherence analysis (Figure 2). Additionally, there was a significantly reduced functional connectivity between the central cingulate gyrus (BA23) and right frontal lobe (BA10) for the gamma 2 frequency band under lagged phase analysis (Figure 3). With other frequency bands there was no significant difference between the two groups.

## Discussion

This study explored changes in central nervous activities in ISSNHL with tinnitus patients when compared with healthy subjects. Overall, in the alpha 2 frequency band, the neural activity of ISSNHL patients with tinnitus was abnormally decreased in the left frontal lobe (BA6). Connections between the temporal gyrus (BA20) and supramarginal gyrus (BA40) as well as between central cingulate gyrus (BA23) and right frontal lobe (BA10) were weakened in the gamma 2 frequency band. This suggests that ISSNHL patients with tinnitus had aberrant brain area activity and functional connectivity.

Source localization analysis found that ISSNHL patients had decreased activity in the left frontal lobe (BA6) in the alpha 2 frequency band, showing similarity to the study by Micarelli et al. (2017), where ISSNHL subjects had a significant decrease of glucose metabolism in the middle frontal gyrus. It is known that the frontal lobe is one of the key areas of the DMN. Under normal circumstances, the regions of the DMN have a raised level of neuronal activity at rest (van den Heuvel and Hulshoff Pol, 2010). Activity and connectivity of the DMN are associated with the core processes of cognition, such as the integration of cognition and processing of emotion (Greicius et al., 2003), monitoring the environment (Gusnard et al., 2001) and mind-wandering (Mason et al., 2007). A study by Zhang et al. (2016) showed that long-term unilateral ISSNHL contributed to alterations of DMN, and that these alterations might influence cognitive abilities in these subjects. In addition, tinnitus as a symptom involving a perceived phantom sound, might lead to dysfunction of DMN (Chen et al., 2017). Our finding of decreased activation of the left frontal lobe might suppose that the DMN was suppressed in resting state, which consequently may activate other brain networks. It might be the reason for patients with attention and emotion issues.

Linear lagged connectivity and lagged coherence showed a weakened connection between the temporal gyrus (BA20) and supramarginal gyrus (BA40) in the gamma 2 frequency band. Previous studies by Fan et al. (2015) showed that unilateral ISSNHL patients had a significant decrease in contralateral auditory gyrus gray matter. A study by Micarelli et al. (2017) suggested a hypometabolism in the contralateral auditory gyrus in ISSNHL patients. The damage to the hair cells in ISSNHL patients causes a lack of auditory signal in the auditory network (van der Loo et al., 2009). These current results are consistent with our previous EEG microstate study (Cai et al., 2019) where there was a reduction of transition from microstate D to A in ISSNHL subjects with tinnitus that correlated with tinnitus scores. Microstate A is related to activation in the bilateral superior and middle temporal gyrus regions that are correlated with phonological processing. The supramarginal gyrus is a part of a dorsal attention network which relates to microstate D. These results indicated that generation of tinnitus in ISSNHL patients may be due to disruption of auditory and non-auditory networks.

Lagged phase synchronization showed that the connection between the central cingulate gyrus (BA23) and right frontal lobe (BA10) was decreased. Both areas are part of the DMN. The DMN is suggested to be particularly active at rest, when no tasks are performed (Mantini et al., 2007). The posterior cingulate node seems to be particularly vital, because it appears to be directly correlated with other nodes of the network and putatively acting as a mediator of intrinsic connectivity across these areas (Rosazza and Minati, 2011). This is likely because it is one of the most intensively interconnected areas in the whole brain (Cavanna and Trimble, 2006). In a task-based scenario, the network deactivated (Raichle and Snyder, 2007). Conversely, a significant increase in fluorodeoxyglucose uptake was seen in the right superior and medial frontal gyrus in ISSNHL patients (Micarelli et al., 2017). This might be a result of heterogeneity between studies. Therefore, more research is needed to understand this difference. In addition, some functional magnetic resonance imaging (fMRI) studies on tinnitus patients found aberrant functional connectivity within the DMN (Burton et al., 2012; Schmidt et al., 2013). The suppression of tinnitus would lead to a differential response pattern among the network nodes. This might result in the inhibiting effect of DMN.

In summary, the findings of our study identify specific inhibition of brain areas and functional connectivity in ISSNHL with tinnitus subjects. The inhibition of the DMN implies that ISSNHL patients have suppressed activity at resting state and are more active in involving attention or emotional behavior when compared with healthy controls. The reduced connection between the auditory system and dorsal attention network suggests decreased attention to auditory information. The brain activity is in a state of inhibition for ISSNHL patients with tinnitus.

Some limitations in this study need to be realized. ISSNHL is a heterogeneous disease. ISSNHL subjects had differences in hearing loss, laterality or accompanying symptoms (van der Loo et al., 2009). So, it was difficult to eliminate the heterogeneous factors completely even using strict inclusion and exclusion criteria. Without specific subgroups of ISSNHL with tinnitus patients, the results of our study need to be read with caution. In addition, the small sample size in the present study is unable to rule out heterogeneous factors. Therefore, we need to explain the results carefully. A further study with larger sample sizes and more subgroup comparisons is needed to investigate the underlying mechanisms in ISSNHL with tinnitus.

## Conclusion

In this study, it was found that the central system of ISSNHL with tinnitus patients had been changed when compared with healthy controls. By using resting-state EEG and analyzing source localization and functional connectivity, we found that ISSNHL and tinnitus patients were abnormally weakened in the left frontal lobe (BA6) in the alpha 2 frequency band. The connection between the temporal gyrus (BA20) and supramarginal gyrus (BA40) is weakened in the Gamma 2 frequency band as well as the connection between the central cingulate gyrus (BA 23) and right frontal lobe (BA 10). These findings could help to make sense of the central pathogenesis in ISSNHL patients.

## Data Availability

There is no other available data. Nonetheless, the data that support the results of the research can be obtained by emailing zhengyiq@mail.sysu.edu.cn.

## Ethics Statement

The studies involving human participants were reviewed and approved by Institutional Review Board of the Sun Yat-sen Memorial Hospital at Sun Yat-sen University of China. The patients/participants provided their written informed consent to participate in this study.

## Author Contributions

The concept and design of this research come from YxC and JL. The experiments were performed by YhC, WC, and WL. CD, GC, SC, and ML analyzed the data. FZ and YZ drafted this manuscript. All authors participated in preparing this manuscript.

## Conflict of Interest Statement

The authors declare that the research was conducted in the absence of any commercial or financial relationships that could be construed as a potential conflict of interest.
